# Entropy as a Robustness Marker in Genetic Regulatory Networks

**DOI:** 10.3390/e22030260

**Published:** 2020-02-25

**Authors:** Mustapha Rachdi, Jules Waku, Hana Hazgui, Jacques Demongeot

**Affiliations:** 1 Team AGIM (Autonomy, Gerontechnology, Imaging, Modelling & Tools for e-Gnosis Medical), Laboratory AGEIS, EA 7407, University Grenoble Alpes, Faculty of Medicine, 38700 La Tronche, France; Mustapha.Rachdi@univ-grenoble-alpes.fr (M.R.); hazgui_hana@yahoo.fr (H.H.); 2 LIRIMA-UMMISCO, Université de Yaoundé, Faculté des Sciences, BP 812 Yaoundé, Cameroun; jules.waku@gmail.com

**Keywords:** genetic regulatory network, complexity, robustness, attractor entropy, isochronal entropy, dynamic entropy, entropy centrality

## Abstract

Genetic regulatory networks have evolved by complexifying their control systems with numerous effectors (inhibitors and activators). That is, for example, the case for the double inhibition by microRNAs and circular RNAs, which introduce a ubiquitous double brake control reducing in general the number of attractors of the complex genetic networks (e.g., by destroying positive regulation circuits), in which complexity indices are the number of nodes, their connectivity, the number of strong connected components and the size of their interaction graph. The stability and robustness of the networks correspond to their ability to respectively recover from dynamical and structural disturbances the same asymptotic trajectories, and hence the same number and nature of their attractors. The complexity of the dynamics is quantified here using the notion of attractor entropy: it describes the way the invariant measure of the dynamics is spread over the state space. The stability (robustness) is characterized by the rate at which the system returns to its equilibrium trajectories (invariant measure) after a dynamical (structural) perturbation. The mathematical relationships between the indices of complexity, stability and robustness are presented in case of Markov chains related to threshold Boolean random regulatory networks updated with a Hopfield-like rule. The entropy of the invariant measure of a network as well as the Kolmogorov-Sinaï entropy of the Markov transition matrix ruling its random dynamics can be considered complexity, stability and robustness indices; and it is possible to exploit the links between these notions to characterize the resilience of a biological system with respect to endogenous or exogenous perturbations. The example of the genetic network controlling the kinin-kallikrein system involved in a pathology called angioedema shows the practical interest of the present approach of the complexity and robustness in two cases, its physiological normal and pathological, abnormal, dynamical behaviors.

## 1. Introduction

The relationships between the notions of complexity, stability and robustness in genetic regulatory networks, as described in [1,2,3], are based on works from the eighties about Boolean automata [4,5,6,7,8,9] on the mathematical characterization of the stability in random dynamical systems. These studies about stability are based on theories of stochastic attractors (called confiners in [10,11]) and large deviations [12,13,14], and on an index of complexity—the evolutionary or dynamic entropy, introduced by L. Demetrius [15,16,17,18,19,20] and complementary to other complexity indices used in biological networks, such as number of nodes, connectivity (i.e., ratio between edge and node numbers) [21,22,23], number of strong connected components of their interaction graph [9], entropy of transformations with invariant measure [24,25], etc. The notion of stability (with respect to robustness) is quantified by the rate at which the system returns to its steady state (with respect to the threshold distance below which the system remains close to its former dynamics in terms of number and nature of attractors) after exogenous and/or endogenous perturbations.

If we restrict the study to discrete dynamical systems, many authors since Rochlin [24,25] have used the notion of entropy for quantifying the stability and robustness of dynamical processes, such as inference in Bayesian networks [26]; symbolic extension of a smooth interval map [27]; symbolic complexity of substitution dynamics on a set of sequences in a finite alphabet [28,29]; algorithmic complexity (called also Kolmogorov-Chaitin complexity) of binary strings involved in mutation dynamics [30]; and eventually, reconstruction and stability of Boolean dynamics of genetic regulatory networks following the René Thomas’ logic rules [31,32,33,34].

In the present paper, we will focus on the study of robustness of genetic regulatory networks driven by Hopfield’ stochastic rule [35], by using the Kolmogorov-Sinaï entropy of the Markov process underlying the state transition dynamics [36,37,38,39,40,41,42,43,44,45,46,47,48,49,50]. In Section 2, we define the concepts underlying the relationships between complexity, stability and robustness in the Markov framework of genetic threshold Boolean random regulatory networks. We give in Section 3 some rigorous results concerning their dynamics, and eventually, in Section 4, we calculate the evolutionary entropy for a particular network controlling the kinin-kallikrein system in physiological and pathological conditions, and we give in Section 5 some perspectives of the present work.

## 2. Materials and Methods

### 2.1. Definitions

The evolutionary (or dynamic) entropy is related to the network dynamics, and can be identified to the Kolmogorov-Sinaï entropy in a Markovian dynamics. Based on the configuration of its attractor (in deterministic case) or confiner (in random case) landscape in the network state space, it is related to the richness of the network attractor or confiner landscape [11]. The literature on network entropies is abundant [19,20,21,22,51,52,53,54,55,56,57,58,59,60,61,62] and concerns both discrete or continuous dynamical systems, which share common mathematical concepts, such as attractor, attraction basin, Jacobian interaction graph, stability and robustness. We take as the definition of an attractor, that given in [63,64], available for both continuous and discrete cases. More specific definitions of the concept of attractor adapted for network dynamics are given in [65,66,67,68].

In the state space E⊂ℝ^n^ provided with a dynamic flow *φ* and a distance d, {*φ*(a,t)}_t__∊T_ denotes the trajectory starting at state a and being in state *φ*(a,t) at time t, where *φ* is a flow on ExT. L(a), the limit set of the trajectory starting in a, is made of the accumulation points of the trajectory {*φ*(a,t)}_t__∊T_, when the time t tends to infinity in the time set T (either discrete, equal to ℕ or continuous, equal to ℝ):

L(a)={y∊E; ∀ ε>0, ∀ t∈T, ∃ s(ε,t) > t, such as d(φ(a,s(ε,t)),y) < ε},

where s(ε,t) is a time (> t) at which the trajectory is in a ball of radius ε centered in y.

Considering a subset A of E, L(A) is the union of all limit sets L(a), for a belonging to A:

$$L(A)=\cup_{a\in A}L\left(a\right)$$

**Figure 1:**
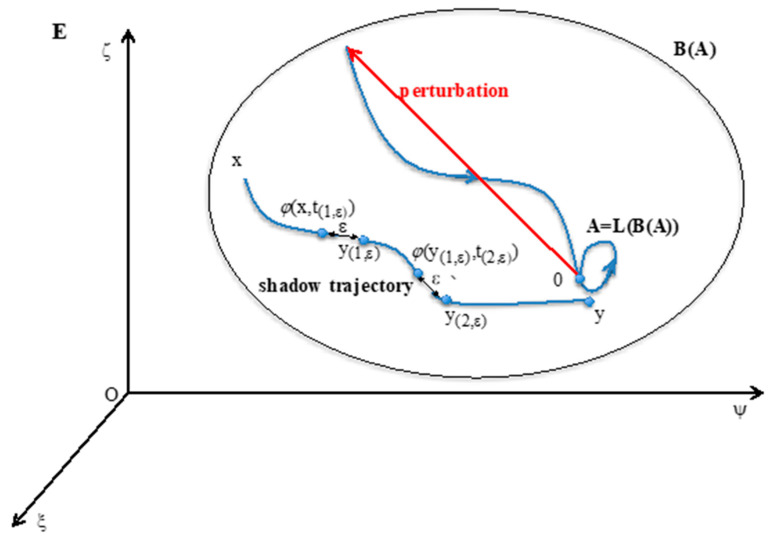
The attractor A is invariant for the operator LoB in the state space E, whose general state is x = (ξ,ψ,ς). The shadow trajectory between x in B(A) and y near A. The point 0 on A is returning to A after a perturbation in its attraction basin B(A).

Conversely, B(A) is the set of all initial conditions (outside A), whose limit set L(a) is included in A (Figure 1). B(A) = {y∊E\A; L(y)⊂A)} is called the attraction basin of A.

An attractor A verifies the three following conditions (Figure 1 Top left):

(i) A = LoB(A).
(ii) There is no set A’ containing strictly A and shadow-connected to A.
(iii) There is no set A” strictly contained in A verifying (i) and (ii).

The definition of the “shadow-connectivity” between subsets F_1_ and F_2_ of E, lies on the fact that there exists a “shadow-trajectory” between F_1_ and F_2_ (cf. Figure 1, where F_1_ = {x} and F_2_ = {y}). The notion of “shadow-trajectory” has been defined first in [69]: for any ε > 0, there is an integer *n*(ε), times t_(1,__ε)_,…, t_(*n*(__ε),__ε)_ and states y_(1,__ε)_,…, y_(*n*__(ε)__,ε)_ = y, such that all distances between successive states of the shadow-trajectory are less than ε:

$$d\left(\varphi \left(x,t_{\left(1,\varepsilon \right)}\right),y_{\left(1,\varepsilon \right)}\right)\leq \varepsilon ,\ldots ,\;d\left(\varphi \left(y_{\left(k-1,\;\varepsilon \right)},t_{\left(k,\varepsilon \right)}\right),y_{\left(k,\varepsilon \right)}\right)\leq \varepsilon ,\ldots ,d\left(\varphi \left(y_{\left(n\left(\varepsilon \right)-1,\;\varepsilon \right)},t_{\left(n\left(\varepsilon \right),\varepsilon \right)}\right),y)\leq \varepsilon \right)$$

For all known dynamical systems, the above definition of attractor remains available, and is meaningful for any discrete or continuous time set T, when the dynamical system is autonomous (with respect to the time), contrary to the other definitions proposed since the seventies, such as in [70,71,72], which are all less general.

### 2.2. Hopfield Dynamics

Let consider now discrete Hopfield dynamics [35], defined in a genetic regulatory network N having n genes, where *x_i_*(*t*) = 1, if the gene i is expressing its protein; else x_i_(t) = 0. The probability that *x_i_*(*t*) = 1, knowing the states at time t in a neighborhood V_i_ of i, is given by:

(1) $$P\left(\left\{x_{i}\left(t\right)=1\right\}|\left\{x_{j}\left(t-1\right),\;j\in V_{i}\right\}\right)=\frac{e^{\left(\sum_{j\in V_{i}}w_{ij}\;x_{j}\left(t-1\right)-\theta \right)/T}}{1+e^{\left(\sum_{j\in V_{i}}w_{ij}\;x_{j}\left(t-1\right)-\theta \right)/T}}$$

*w_ij_* is a parameter representing the influence or weight (inhibitory, if *w_ij_* < 0; activating, if *w_ij_* > 0 and null, if *w_ij_* = 0) the gene *j* from the neighborhood *V_i_* ={*j*; *w_ij_* ≠ 0} exerts on the gene *i*, and which corresponds to the notion of external field in statistical mechanics and here to the minimal value of activation for interaction potential: $\sum_{j\in V_{i}}w_{ij}\;x_{j}$.

The thermodynamic parameter, temperature *T*, allows for introducing a certain degree of randomness in the transition rule. In particular, if *T* = 0, the rule is practically deterministic:

$$x_{i}\left(t\right)=1,\;if\;\sum_{j\in V_{i}}w_{ij}\;x_{j}\left(t-1\right)>\theta ;\;x_{i}\left(t\right)=0,\;if\;\sum_{j\in V_{i}}w_{ij}\;x_{j}\left(t-1\right)<\theta$$

(2) $$\Leftrightarrow \;x_{i}\left(t\right)=H\left(\sum_{j\in V_{i}}w_{ij}\;x_{j}\left(t-1\right)-\theta \right)$$

and

$$P\left(\left\{x_{i}\left(t\right)=1\right\}|\left\{x_{j}\left(t-1\right),\;j\in V_{i}\right\}\right)=\frac{1}{2},\mathrm{if}\;\sum_{j\in V_{i}}w_{ij}\;x_{j}\left(t-1\right)=\theta$$

where *H* is the Heaviside function.

Once this rule defined, three incidence matrices of important graphs characterizing the network dynamics can be defined:

- Interaction matrix *W*: Its coefficient *w_ij_* corresponds to the action of the gene *j* on the gene *i*. *W* is analogue to a discrete Jacobian matrix. Signed Jacobian matrix *A* is defined as follows: *α_ij_*=1, if *w_ij_* > 0, *α_ij_* = −1, if *w_ij_* > 0 and *α_ij_*=0, if *w_ij_*=0. Its associated directed graph is the interaction graph *G*;
- Updating matrix *S*: *s_ij_* = 1, if j is updated before or with i, else *s_ij_* = 0;
- Flow matrix *Φ*: *ϕ_bc_* = 1, where *b* and *c* belong to E, if and only if *b* = *φ*(*c*,1); else *ϕ_bc_* = 0.

**Figure 2:**
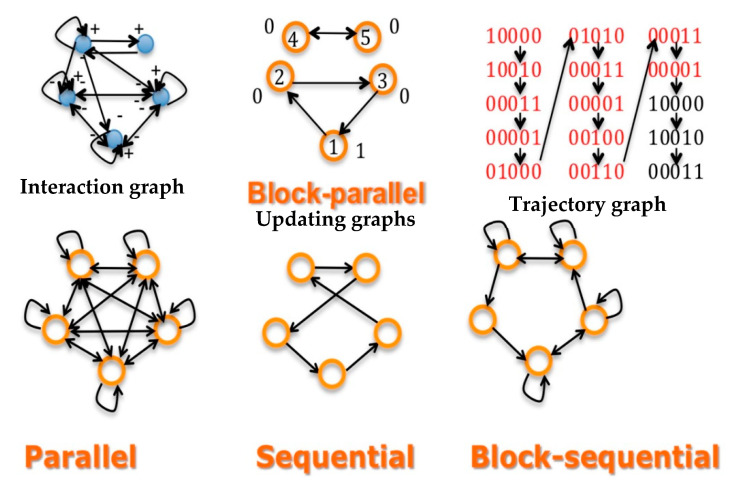
Top left: interaction graph *G* of a network made of a 3-switch linked to a regulon representing a genetic clock. Top middle: the updating graph corresponding to block-parallel dynamics ruling the network. Top right: a part of the trajectory graph of the dynamics exhibiting a limit-cycle of period 12 having internally a cycle of period 4 for the clock. Bottom: updating graphs corresponding successively (from the left to the right) to the parallel, sequential and block-sequential dynamics.

The matrices involved in the network dynamics are constant or depend on time t [41,42]:

- Concerning *W*, the dependence is called the Hebbian dynamics: if the two vectors {*x_i_*(*s*)}*_s_*_<*t*_ and {*x_j_*(*s*)}*_s_*_<*t*_ have a correlation coefficient *j*(*t*)≠0, then the dependence is expressed through the equation: *w_ij_*(*t* + 1) = *w_ij_*(*t*) + *h_ij_*(*t*), with *h* > 0, corresponding to a reinforcement of the absolute value of interactions *w_ij_*(*t*) having succeeded to increase the *x*i(*s*)’s, if *w_ij_*(*t*) is positive, and conversely to decrease the *x_i_(s*)’s, if *w_ij_*(*t*) is negative.
- Concerning S, the updating can be state dependent or not. If all *s_ij_* equal one, the updating schedule is called parallel; if there exists a sequence of indices of the n nodes of the network, *i*_1_,…, such as *s_i__k__i__k_*_+1_ = 1, the other *s_ij_*’s being equal to 0, the updating is called sequential. Between these two extreme schedules, the updating modes are called block-sequential, i.e., they are parallel in a block, the blocks being updated sequentially. A more realistic schedule in genetic and metabolic networks is called block parallel [50]: These networks are composed of blocks made of genes sequentially updated, these blocks being updated in parallel (Figure 2 Top middle). Some interactions between these blocks can exist (i.e., there are *w_ij_* ≠ 0, with *i* and *j* belonging to 2 different blocks), but because the block sizes are different, the time at which the first attractor state is reached in a block is not necessarily synchronized with the corresponding times in other blocks: the synchronization expected as asymptotic behavior of the dynamics depends on the intra-block as well as on the inter-block interactions, which explains that states of genes in a block serving as the clock for the network are highly dependent on states of genes in other blocks connected to them (e.g., acting as transcription factors of the clock genes).
- Concerning *Φ*, the transition operator is Markov, and hence autonomous in time.

Figure 2 Top shows the three important graphs of the network dynamics; i.e., the graphs having as incidence matrices respectively, the interaction W (left), updating S (middle) and trajectory (right) matrices. If the dynamics are ruled by a deterministic Hopfield network, with temperature equal to 0 and interaction weights *w_ij_*, which are equal to 1, −1 or 0, their attractor is a limit-cycle given on Figure 2 (top right), showing two intricate rhythms, one of Period 4 corresponding to the inner clock dynamics, embedded in a rhythm of period 12, the rhythm of the whole network. Figure 2 (Bottom) shows other updating graphs, less realistic than the block-parallel for representing the clock action.

## 3. Results

### 3.1. Attractor Entropy and Isochronal Entropy, Indices of Complexity and Synchronizability

The attractor entropy *E_attractor_* is a measure of heterogeneity of the attractor landscape on the state space E of a dynamical system. It is defined by the quantity:

(3) $$E_{attractor}=-\sum_{k=1,m}ABRS\left(A_{k}\right)log_{2}\left[ABRS\left(A_{k}\right)\right],$$

where *ABRS*(*A_k_*) is equal to the attraction basin relative size of the *k*th attractor *A_k_* among the *m* attractors of the network dynamics, divided by 2*^n^*, *n* being the number of genes of the network. Let consider the case of a continuous network, called the Wilson-Cowan oscillator, close to the Hopfield system if *τ*_x_ = *τ*_y_ = 1/ε, *w_xx_* = *w_xy_* = *w_yx_* = *w_yy_* = *λ*/*ε*, 0<*ε*<<1 [73], which describes the activities *x* and *y* of two populations of interacting genes as follows:

$$\frac{dx}{dt}=-\frac{x}{\tau_{x}}+\tan h\left(w_{xx}x-\theta \right)-\tan h\left(w_{xy}y-\theta \right)$$

(4) $$\frac{dy}{\;dt}=-\frac{y}{\tau_{y}}+\tan h\left(w_{yx}x-\theta \right)+\tan h\left(w_{yy}y-\theta \right).$$

The variables x and y represent the proteinogenic activities of two populations of genes, whose interaction is described through a quasi-sigmoid function parametrized by *λ*, controlling its stiffness and accounting for the response to a signal coming from the other gene (whose expressed protein acts as a transcription factor, inhibitor or activator, on the first gene). This response is highly non-linear when *λ* is large, becoming similar to a Hopfield-like response. 

**Figure 3:**
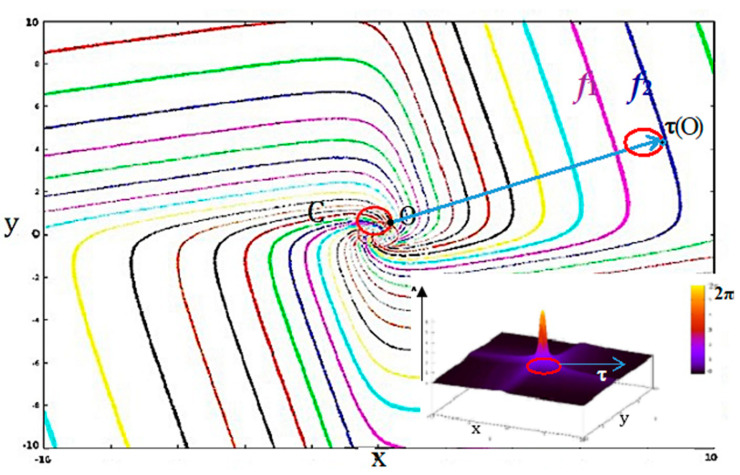
Isochrons and maximum phase shift of the Wilson-Cowan oscillator. In the phase space (x0y) are 30 isochrons and the limit cycle C (in red) of a Wilson-Cowan oscillator (with *τ_x_* = *τ_y_* = *τ* = 1, and *λ* = 1.1). The oscillation period is 2π in the vicinity of its Hopf bifurcation; the limit cycle C is close to the unit circle; and isochrons are in the form of spirals. Thumbnail Bottom Right: representation of the maximum phase shift along the z axis with a color code (between 0 and 2π). The profile of maximum phase shift indicates how a population of Wilson-Cowan oscillators synchronizes following an instantaneous translation *τ*, and the degree of synchronization (inverse of this maximum phase shift observed after perturbation) is proportional to the intensity of the translation.

The parameters *τ_x_* and *τ_y_* refer to the influence power of the populations of genes *x* and *y*. If θ = 0, *τ_x_ = τ_y_ = τ*, the Wilson-Cowan oscillator presents a Hopf bifurcation when *λτ* crosses the value 1 [74], moving from dynamics with only one stable fixed point to dynamics with a repulsor at the origin of the state space E, surrounded by an attractor limit cycle C of period *T*. Let us consider now the point *φ*(O,*h*) of C reached after a time (or phase) *h* on C from a point chosen as origin O = *φ*(O,0) (Figure 3): there is a bijective map *b* between C and the phase interval [0, *T*]. The isochron *I_h_* of phase *h* is the set of points *x* of B(C) such that d(*φ*(*x*,*t*), *φ*(*h*,*t*)) tends to 0, when *t* tends to infinity. *I_h_* extends the map *b* to all the points of B(C). Let denote *φ_h_* the flow *φ* restrained to *I_h_*xT_d_, where T_d_ = {k*T*}_k∈ℕ_, such as:

$$\forall x\in E,\forall t\in T_{d},\;\mathrm{then}\;\varphi_{h}\left(x,t\right)=\varphi \left(x,t\right)\in I_{h},\;\mathrm{if}\;h=lim_{t\in T_{d};\;t\to \infty }\varphi \left(x,t\right)\;\;\mathrm{and}\;B\left(C\right)=\cup_{h\in C}I_{h}$$

The point *h* is an attractor for *φ_h_*, whose basin is *I_h_*\{*h*}. Let us decompose now the time interval [0,*T*] in *m* equidistant sub-intervals [k*T*/*m*, (k+1)*T*/*m*[, for k = 0,…,*m*−1. Let denote *φ*_k_ the flow *φ* restrained to A_k_∪B_k_ x T_d_, with A_k_ = F([k*T*/*m*, (k+1)*T*/*m*]) as attractor, having as attraction basin the set B_k_ = ∪*_h_*_∊F([k*T*/*m*, (k+1)*T*/*m*[)_ *I_h_*, with B(C) = ∪_k=0,*m*−1_B_k_. The isochronal entropy of degree *m* is related to the *m* A_k_’s subsets of of C=∪_k=0,*m*−1_A_k_, each A_k_ being the attractor for the dynamics *φ*_k_:

(5) $$E_{attractor}^{m}=-\sum_{k=1,m}ABRS\left(A_{k}\right)log_{2}\left[ABRS\left(A_{k}\right)\right]$$

If isochron basins have all the same size, then *E^m^_attractor_ =* log_2_*m*. If not, *E^m^_attractor_* reflects the spatial heterogeneity of the asymptotic behavior of the network dynamics: the basins B_k_ can be large (when the flow *φ* is rapid inside) or small (when the flow *φ* is slow inside). In the example of the Wilson-Cowan oscillator with *τ_x_ = τ_y_* = 1, if *λ* is growing from 0 to infinite, then *E^m^_attractor_* is increasing from 0 to log_2_*m* [75].

Let us fix now the values of the parameters: *τ_x_* = *τ_y_* = *τ* = 1 and *λ* = 1.1, close to the bifurcation in the parameter space (*λτ* ≈ 1) and consider the isochron landscape of the Wilson-Cowan oscillator (Figure 3). The maximum phase shift observed after an instantaneous translation of the limit cycle of the Wilson-Cowan oscillator is inversely proportional to the intensity of this perturbation (Figure 3 Thumbnail), which means that a population of oscillators must be perturbed by a sufficiently intense stimulus to be synchronized. Indeed, isochrons form spirals, which diverge from one another far from the limit cycle and the synchronization, are observed if the translation of the limit cycle falls between two isochrons having close phases *f*_1_ and *f*_2_. These isochrons can be reached by translating the limit cycle C sufficiently far from its initial position [75,76,77]: it is possible if attraction basins are sufficiently wide, and hence, if the isochronal entropy *E^m^_attractor_* is sufficiently large. Hence, if *E_attractor_* can serve as index of complexity of attractor landscape, *E^m^_attractor_* can serve more precisely as index of synchronizability, which is particularly interesting in the study of biological clocks and the entrainment of biological rhythms.

### 3.2. Energy, Frustration and Dynamic Entropy

We define first the functions energy *U* and frustration *F* for a genetic network *N* with *n* genes in interaction [1,2,15,16,17,18,19,20,21,22,23,36,37,38,39,40,41,42,43,44,78]:

(6) $$\forall x\in E,\;U\left(x\right)=\sum_{i,j=1,n}\alpha_{ij}x_{i}x_{j}=Q_{+}\left(N\right)-F\left(x\right),$$

where *x* is a configuration of gene expression (*x_i_* = 1, if the gene *i* is expressed, and *x_i_* = 0, if not), E denotes the set of all configurations of gene expression; that is, for a Boolean network, the hypercube {0,1}*^n^*, and *α_ij_* = sign(*w_ij_*) is the sign of the interaction weight *w_ij_*, which quantifies the influence of the gene *j* on the gene *i*: *α_ij_* = −1 (with respect to +1), if *j* is an inhibitor (with respect to activator) of the expression of *i*, and *α_ij_* = 0, if *j* exerts no influence on *i*. Q_+_(*N*) is equal to the number of positive edges of the interaction graph *G* of the network *N* having *n* genes, whose incidence matrix is *A* = (*α_ij_*)*_i_*_,*j* = 1,*n*_. *F*(*x*) denotes the global frustration of *x*; i.e., the number of pairs of genes (*i*,*j*) for which state values *x_i_* and *x_j_* are contradictory with the sign *α_ij_* of the influence of *j* on *i*:

(7) $$F\left(x\right)=\sum_{i,j=1,n}F_{ij}\left(x\right),$$

where *F_ij_* is the local frustration of the pair (*i*,*j*) defined by:

*F_ij_*(*x*) = 1, if {*α_ij_* = 1 ∧ {{*x_j_* = 1 ∧ *x_i_* = 0}∨{*x_j_* = 0 ∧ *x_i_* = 1}} ∨ {*α_ij_* = −1 ∧ {{*x_j_* = 1 ∧ *x_i_* = 1}∨{*x_j_* = 0 ∧ *x_i_* = 0}}, else *F_i_*_j_(*x*) = 0.

We choose for the dynamics of the network the Hopfield rule (1). It corresponds to a Markov operator defined on the state space E provided with a sequential updating schedule for the expression of the genes in a predefined order [1,2]. Let *M* = (*M_xy_*) denote its Markov matrix, giving the transition probability (defined by the rule (1) and this sequential updating mode) to go from the configuration *x* to the configuration *y* in E, and *μ* = {*µ_x_* = *μ*({*x*})}*_x_*_∊__E_ denote its stationary distribution on E. The dynamic entropy *E* is then defined as the Kolmogorov-Sinaï entropy of *M* by the classical formula:

(8) $$E=-\sum_{x,y\in E}\mu_{x}M_{xy}log_{2}\left(M_{xy}\right)$$

In the sequential updating mode defined by the order of the nodes equal to the integer order on *N*={1,*n*}, by denoting *I* = {1,…,*i* − 1}, *N\I* = {*i*,…,*n*} and *X* (with respect to *Y*) the set of the indices *i* such that *x_i_* = 1 (with respect to *y_i_* = 1), we have for the general coefficient of the transition matrix *M*:

(9) $$M_{xy}=\prod_{i=1,n}[P\left(\left\{y_{i}=1\right\}|\left\{y_{j},\;j\in I;\;x_{k},\;k\in N\backslash I\right\}\right)I_{\left\{y_{i}=1\right\}}\;+P\left(\left\{y_{i}=0\right\}|\left\{y_{j},\;j\in I;\;x_{k},\;k\in N\backslash I\right\}\right)I_{\left\{y_{i}=0\right\}}]$$

and for the invariant measure, the classical Gibbs measure *µ* [1,2]:

(10) $$\forall x\in E,\;\mu_{x}=\frac{e^{\left(\sum_{i,j=1,n}w_{ij}\;x_{i}x_{j}-\theta \right)/T}}{\sum_{y\in E}e^{\left(\sum_{i,j=1,n}w_{ij}\;y_{i}y_{j}-\theta \right)/T}}$$

**Figure 4:**
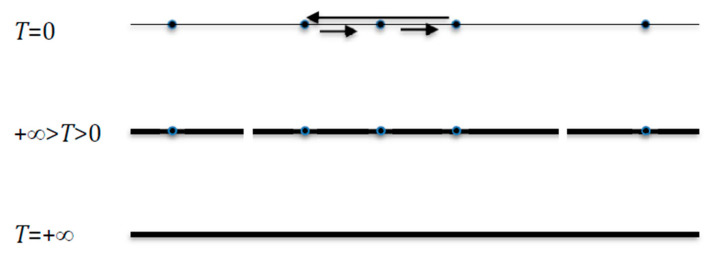
The invariant measure *μ* is scattered uniformly. Top: if *T* = 0, over deterministic attractors (two fixed points and a limit cycle). Middle: if +∞ > *T* > 0, over attraction basins of these attractors, considered as final classes of the Markov transition matrix. Bottom: if *T* = +∞, over the whole state space E.

When *T* = 0, the invariant measure *μ* is concentrated on the *m* (≤2*^n^*) deterministic attractors *A_k_* of size *a_k_* of the network dynamics (e.g., two fixed points and a limit cycle on Figure 4 Top) and we have:

(11) $$E=-\sum_{x,y\in E}\mu_{x}M_{xy}log_{2}\left(M_{xy}\right)=0,$$

because each line of *M* is reduced to one coefficient equal to one, the others being equal to 0.

When +∞ > *T* > 0, if *μ* becomes scattered uniformly on the attraction basins of the *m* deterministic attractors supposed to be fixed points of the network dynamics (cf. Figure 4 Middle), they are transiently the final classes of the Markov transition matrix related to the dynamics (9), and are denoted *A_k_* of size card(*A_k_*) = *a_k_*. Then we have, when this scattering is observed, the approximate formula:

$$E=-\sum_{x,y\in E\;}\mu_{x}M_{xy}log_{2}M_{xy\;}\approx -\sum_{k=1,m}\frac{a_{k}}{2^{n}}log_{2}\left(\frac{1}{2^{n}}\right)\;=log_{2}\left(2^{n}\right)+\sum_{k=1,m}\frac{a_{k}}{2^{n}}log_{2}\left(\frac{a_{k}}{2^{n}}\right)$$

which can be written as:

(12) $$E\approx log_{2}\left(2^{n}\right)-\sum_{k=1,m}ABRS\left(A_{k}\right)log_{2}\left[ABRS\left(A_{k}\right)\right]=log_{2}\left(2^{n}\right)-E_{attractor}$$

where *ABRS*(*A*) = Σ*_x_*_∊_*_B_*_(*A*)__∪_*_A_* *μ_x_* = *μ*(*B*(*A*)∪*A*).

When *T* tends to +∞, *μ* tends to be scattered uniformly over E (cf. Figure 4 Bottom) and *E* = log_2_2*^n^* = *n*. More generally, we have:

(13) $$E=-\sum_{x,y\in E\;}\mu_{x}M_{xy}log_{2}M_{xy\;}=-\sum_{x,y\in E\;}\mu_{x}M_{xy}log_{2}(\mu_{x}M_{xy\;})+\mu_{x}log_{2}\mu_{x}\;=E_{\nu }-E_{\mu }$$

where *E_v_* and *E_µ_* denote, respectively, the entropy of the joint measure $\nu =\mu_{x}M_{xy}$ on E^2^ and the entropy of the invariant measure *µ*, which can be estimated by *E_attractor_*, if attractors are fixed points and by *E^m^_attractor_* if the attractor is a limit-cycle. 

When *T* = +∞, and *v* and *μ* are scattered uniformly, respectively, on E^2^ and E, and we have: *E* = log_2_ ((2*^n^*)^2^)−log_2_2*^n^* = *n*.

When *T* = 0, if *v* is concentrated on a set of states in E^2^ and *µ* is concentrated on the projection of this set of states on E, then they have the same entropy and E = 0. In between (when +∞ > *T* > 0), Equation (12) constitutes an estimator of *E*, if *T* is s large enough.

### 3.3. Dynamic Entropy, Index of Robustness

The problem of robustness of a biological network controlling important functions, such as morphogenesis, memory, cell division, etc., has been often considered for 80 years [79,80,81] under different names (structural stability, resilience, bifurcation, etc.), but is still pertinent [82,83].

The dynamic entropy *E* serves as index of robustness, being related (as Kolmogorov-Sinaï entropy) to the ability a system has to return to its equilibrium after exogenous or endogeneous perturbations [23,43].

By considering a Hopfield rule with all non-zero interaction weights *w_ij_* having the same absolute value *c*, we will study the robustness of the network in response to the variations of *c*, by proving the following Propositions [39,42]:

**Proposition** **1.** *Let us consider a deterministic Hopfield Boolean network, which is a circuit sequentially or synchronously updated with constant absolute value c for its non-zero interaction weights. Then, its dynamics are conservative, keeping constant on the trajectories the Hamiltonian function L defined by:*

(14) $$L\left(x\left(t\right)\right)=\sum_{i=1,n}\frac{{\left(x_{i}\left(t\right)-x_{i}\left(t-1\right)\right)}^{2}}{2}=\sum_{i=1,n}\frac{H{\left(w_{i\left(i-1\right)modn}x_{i-1}\left(t-1\right)-x_{i}\left(t-1\right)\right)}^{2}}{2}$$

*whereHdenotes the classical Heaviside function. L*(*x*(*t*)) *is the total discrete kinetic energy, equal to the half of the global dynamic frustrationF*(*x*(*t*)) = ∑*_i_*_=1,*n*_*F_i_*_,(*i*−1)modn_ (*x*(*t*)), *where* *F_i_*_,(*i*−1)modn_ *is the local dynamic frustration defined between nodes* (*i*−1) *and* *i* *as follows:*

*F_i_*_,(*i*−1)_(*x*(*t*)) *= 1, if {sign*(*w_i_*_(*i*−1)_) = *1 ∧x_i_*(*t*) ≠ *x_i_*_−__1_(*t* *− 1)} ∨ {sign*(*w_i_*_(*i*−1)_) = −1 ∧ *x_i_*(*t*) = *x_i_*_−__1_(*t* − 1)}, *else* *F_i_*_,(*i*−1)_(*x*(*t*)) = 0.

**Figure 5:**
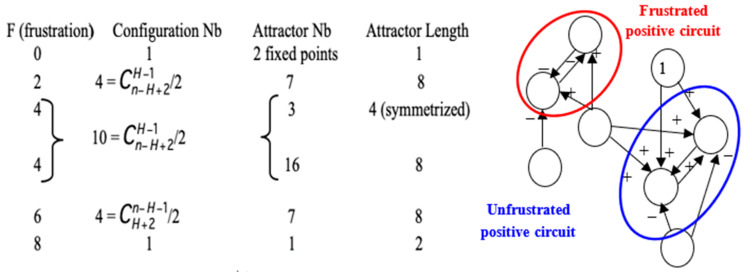
**Left**: Description of the attractors of circuits of length eight for which Boolean local transition functions are either identity or negation. **Right**: Frustrated pair of nodes belonging to a positive circuit of length two in the genetic network controlling the flowering of *Arabidopsis thaliana*. The network evolves by diminishing the global frustration until the attractor on which the global frustration remains constant.

The Proposition 1 still holds if the network is a circuit whose transition functions are Boolean identity or negation, a circuit on which it is easy to calculate the global frustration *F* and to show that it characterizes attractors by remaining constant along them and decreasing inside their attraction basin like in discrete gradient dynamics (Figure 5 Left) [84].

Let consider now the quantity ∂*E*/∂*c*, which denotes the derivative of the dynamic entropy *E* with respect to the common absolute value *c*. ∂*E*/∂*c* can be considered as the capacity the robustness parameter *E* has to resist to environmental variations of the network weights and we have from formula (13): ∂*E*/∂*c* = ∂*E_v_*/∂*c* − ∂*E_µ_*/∂*c.* Then we can prove the following result [29,42]:

**Proposition** **2.** *If the updating mode of the Hopfield network is sequential, we have:*

∂*E_µ_*/∂*c* = −*c***Var***_µ_*(*F*), (15)

*where the variance **Var**_µ_ is taken for the invariant Gibbs measure µ defined by:*

$$\forall \;x\in E,\;\mu_{x}=\frac{e^{\left(\sum_{i,j=1,n}w_{ij}\;x_{i}x_{j}-\theta \right)/T}}{\sum_{y\in E}e^{\left(\sum_{i,j=1,n}w_{ij}\;y_{i}y_{j}-\theta \right)/T}}$$

**Proof:**  We have:

*E_µ_* = −Σ*_x__∊_*_E_*μ_x_*log_2_*µ_x_*, then ∂*E* /∂*c* = −Σ*_x__∊_*_E_ ∂*μ_x_*/∂*c* log_2_*µ_x_* − Σ*_x__∊_*_E_*μ_x_* ∂log_2_*µ_x_*/∂*c*,

where ∂*μ_x_*/∂*c* = ∂[exp((Σ*_i_*_,*j*=1,*n*_ *cα_ij_x_i_x_j_* − θ)/*T*)/*Z*]/∂*c*; *N_i_* is the neighborhood of *i* made of the nodes *j* such as *α_ij_* ≠ 0, *Z* = Σ*_y_*_∊__E_ exp((Σ*_i_*_,*j*=1,*n*_ *cα_ij_y_j_y_j_* − θ)/*T*) and ∂*Z*/∂*c* = Σ*_y_*_∊__E_ (Σ*_i_*_,*j*=1,*n*_ *α_ij_y_i_y_j_*/*T*)*Zμ_y_*. Then:

∂*μ_x_*/∂*c =*[∂[exp((Σ*_i_*_,*j*=1,*n*_*cα_ij_x_i_x_j_*-θ)/*T*)/∂*c*]/*Z* − exp((Σ*_i_*_,*j*=1,*n*_*cα_ij_x_i_x_j_*-θ)/*T*)(∂*Z*/∂*c*)/*Z*^2^ = (Σ_*i,j*=1,*n*_*α_ij_x_i_x_j_/T*)*μ_x_* − Σ*_y_*_∊__E_ (Σ_*i,j*=1,*n*_*α_ij_y_i_y_j_/T)μ_y_μ_x_*,

which implies:

∂μ_x_/∂c log_2_µ_x_=[c(Σ_i,j=1,n_ α_ij_x_i_x_j_/T)^2^μ_x_ − Σ_y__∊__E_ c(Σ_i,j=1,n_ α_ij_y_i_y_j_/T)(Σ_i,j=1,n_ α_ij_x_i_x_j_/T)μ_y_μ_x_]/Log2 − ∂μ_x_/∂c log_2_Z

and

*μ_x_* ∂log_2_*µ_x_*/∂*c* = (∂*μ_x_*/∂*c*)/Log2

Hence, we have:

∂*E_µ_*/∂*c* = −Σ*_x__∊_*_E_ ∂*μ_x_*/∂*c* log_2_*µ_x_* − Σ*_x__∊_*_E_*μ_x_* ∂log_2_*µ_x_*/∂*c*

= −c[**E***_µ_*(*U*^2^) − (**E***_µ_*(*U*))^2^]/Log2 + Σ*_x__∊_*_E_ [∂*μ_x_*/∂*c* log_2_*Z* − ∂*µ_x_*/∂*c*]/Log2,

but Σ*_x__∊_*_E_ ∂*μ_x_*/∂*c* = ∂(Σ*_x__∊_*_E_ *μ_x_*)/∂*c* = 0, therefore ∂*E_µ_*/∂*c* = −*c***Var***_µ_*(*U*) = −*c***Var***_µ_*(*F*) □

Let us define now the local cross-frustration *G_ij_*(*x,y*) = 1: if *α_ij_* = 1, *x_i_y_j_* = 0 or *α_ij_* = −1, *x_i_y_j_* = 1, and *G_ij_*(*x*,*y*) = 0 elsewhere, the global cross-frustration is *G*(*x,y*) = Σ*_i_*_,*j*=1,*n*_ *G_ij_*(*x,y*). We can remark than *G*(*x*,*x*) = *F*(*x*). The local *V*(*x*,*y*) and conditional *V*(*x*) cross-energy functions are respectively defined as:

*V*(*x*,*y*) = Σ*_i_*_,*j*=1,*n*_*α_ij_x_i_y_j_*/*T* = *Q*_+_(*N*) – *G*(*x*,*y*) and *V*(*x*) = Σ*_y_*_∊__E_*V*(*x*,*y*).

By considering the conditional entropy *E_x_* = −Σ*_y__∊_*_E_ *M_xy_* log_2_*M_xy_*, where, in the parallel updating mode:

(16) $$\;M_{xy}=\prod_{i=1,n}[P\left(\left\{y_{i}=1\right\}|\left\{x\right\}\right)I_{\left\{y_{i}=1\right\}}+P\left(\left\{y_{i}=0\right\}|\left\{x\right\}\right)I_{\left\{y_{i}=0\right\}}]\;=\;\exp((\Sigma_{i,j=1,n}c\alpha_{ij}x_{i}y_{j}-\theta )/T)/\Sigma_{y∊E}\exp((\Sigma_{i,j=1,n}c\alpha_{ij}x_{i}y_{j}-\;\theta )/T)$$

and the invariant measure *µ_x_* equals:

*μ_x_* = Σ*_y_*_∊__E_ exp((Σ*_i_*_,*j*=1,*n*_*cα_ij_x_i_y_j_* − θ)/*T*)/Z, with Z = Σ*_x_*_,_*_y_*_∊__E_ exp((Σ*_i_*_,*j*=1,*n*_*cα_ij_x_i_y_j_* − θ)/*T*) (17)

Then, the following Propositions holds:

**Proposition** **3.** *In parallel updating mode, we have: ∂E_x_/∂c = −c**Var**_x_G, where the variance **Var**_x_ is taken for the conditional measure {M_xy_}_y__∊__E_*.

**Proof:**  It is the same proof as for Proposition 2 by replacing *F* with *G*. □

Let remark now that dynamic entropy *E* is related to the conditional entropy *E_x_* as follows:

(18) $$E\;=\;\sum_{x\in E}\mu_{x}E_{x}$$

**Proposition** **4.** *In parallel updating mode, we have: ∂E/∂c = -c**Var*** *_v_G + **Cov*** *_v_(J,G), were **Var*** *_v_ and **Cov*** *_v_ are respectively the variance and the covariance taken for the random variables G(x,y) and J(x,y) = log_2_* *M_xy_ and for the joint measure v =* *μ_x_M_xy_.*

**Proof:**  We have: ∂*E*/∂*c* = Σ*_x__∊_*_E_ (*μ_x_* ∂*E_x_*/∂*c* + *E_x_* ∂*μ_x_*/∂*c*). Hence, from Proposition 3, we get:

∂*E*/∂*c* = −*c***Var***_v_G* + Σ*_x__∊_*_E_*E_x_* ∂*μ_x_*/∂*c*,

With ∂*μ_x_*/∂*c* = [Σ*_y_*_∊_*_Ω_*[∂exp((Σ*_i_*_,*j*=1,*n*_ *cα_ij_x_i_y_j_*-θ)/*T*)/∂*c*]/*Z* − [Σ*_y_*_∊__E_ exp((Σ*_i_*_,*j*=1,*n*_ *cα_ij_x_i_y_j_* − θ)/*T*](∂*Z*/∂*c*)/*Z*^2^, where *Z* = Σ*_x_*_,_*_y_*_∊__E_ exp((Σ*_i_*_,*j*=1,*n*_ *cα_ij_x_i_y_j_* − θ)/*T*) and ∂*Z*/∂*c* = Σ*_x_*_,_*_y_*_∊__E;_ *_i_*_,*j*=1,*n*_ *α_ij_x_i_y_j_*/*T*)exp((Σ*_i_*_,*j*=1,*n*_ *cα_ij_x_i_y_j_* − θ)/*T*)

Then, we have:

∂μ_x_/∂c = Σ_y__∊__E; i,j=1,n_ α_ij_x_i_y_j_ exp((Σ_i,j=1,n_ cα_ij_x_i_y_j_ − θ)/T)/ZT − μ_x_Σ_x,__y__∊__E; i,j=1,n_ α_ij_x_i_y_j_ exp((Σ_i,j=1,n_ cα_ij_x_i_y_j_ − θ)/T)/ZT = *μ_x_* (**E***_x_*(*G*) – **E***_v_*(*G*))

and Σ*_x_*_∊__E_ *E_x_* ∂*μ_x_*/∂*c* = Σ*_x_*_∊__E_ *μ_x_ E_x_* **E***_x_*(*G*) – *E* **E***_µ_*(*G*) = **E**_v_(*JG*) – **E**_v_(*J*)**E**_v_(*G*).

Finally, we get: ∂*E*/∂*c* = −*c***Var***_v_G* + **Cov***_v_*(*J*,*G*) □

We have shown in the Propositions above a direct link between the sensitivity to *c* of entropies *E_µ_*, *E_x_* and *E*, and the variability of frustrations of the network; e.g., if the network is sequentially updated, *E_µ_* decreases when the variance of the global frustration *F* increases, because of Equation (15): ∂*E_µ_*/∂*c* = −*c***Var***_µ_F* [39].

Remarks:

(1) The Proposition 2 still holds when we replace c, the absolute value of weights *w_ij_*, by the temperature *T*, and we have: ∂*E_µ_*/∂*T* = *Var_µ_(F)/T^3^.*
(2) The Proposition 2 shows that there is a close relationship between the robustness (or structural stability) with respect to variations of the parameter *c* and the global dynamic frustration *F*. This global dynamic frustration *F* is in general easy to calculate. For the configuration *x* of the genetic network controlling the flowering of *Arabidopsis thaliana* [37], there is only one frustrated pair; hence, *F*(*x*) = 2 (Figure 5 Right). More generally, all the thermodynamic functions introduced here are calculable in theory and could serve to quantifying the trajectory (Lyapunov) stability and the structural stability. If the number of states of a discrete state space E is too large, or if integrals on a continuous state space E are too difficult to calculate, it is possible to use Monte Carlo procedures for getting the values of the variance of the global frustration and of the dynamic entropy.

### 3.4. Entropy Centrality of Nodes

There are four classical types of centrality in an interaction graph *G* (Figure 2 Top). The betweenness centrality [85] is the first type and is defined for a node *k* as follows:

(19) $$C_{k}^{b}=\underset{i\neq j\neq k\in G}{\sum }\frac{\beta_{ij}\left(k\right)}{\beta_{k}},$$

where β*_ij_*(*k*) is the number of shortest paths going from *j* to *i* through *k*, and β*_k_* = Σ*_I_*_≠ *j*_*_∊__G_* β*_ij_*(*k*).

The degree centrality is the second type of classical centrality. It is defined from the notions of in-, out- or total-degree of a node *i*, which correspond respectively to the number of arrows of the interaction graph *G* having *i* respectively as end, start, or as both. For example, the in-degree centrality is defined by:

(20) $$C_{i}^{in-deg}=\frac{\sum_{j=1,n}\left|a_{ij}\right|}{n-1}$$

where *a_ij_* denotes the general coefficient of the signed incidence matrix *A* of the graph *G*. 

The closeness centrality is the third type of classical centrality. Closeness, as the inverse of the farness, is defined by considering the inverse of an average distance between *i* and *j*, over all nodes *j* of the interaction graph *G*:

(21) $$C_{i}^{clo}=\frac{n-1}{\sum_{j=1,n}l\left(i,j\right)}$$

where the distance chosen here is *l*(*i*,*j*), the length of the closest path between *i* and *j*.

The eigen-centrality or spectral centrality is the last classical centrality. It takes into account the fact that neighbors of a node *i* are possibly highly connected to the interaction graph, considering that connections to (possibly few) highly connected nodes contribute to the centrality of *i* as much as connections to (possibly numerous) weakly connected nodes. Hence, the eigenvector centrality *C_i_^eigen^* of the gene *i* measures more the global influence of *i* on the whole network and verifies [86]:

(22) $$C_{i}^{eigen}=\lambda \sum_{j=1,n}\;C_{j}^{eigen}$$

where *λ* is the greatest eigenvalue of the incidence matrix of the interaction graph *G*.

**Figure 6:**
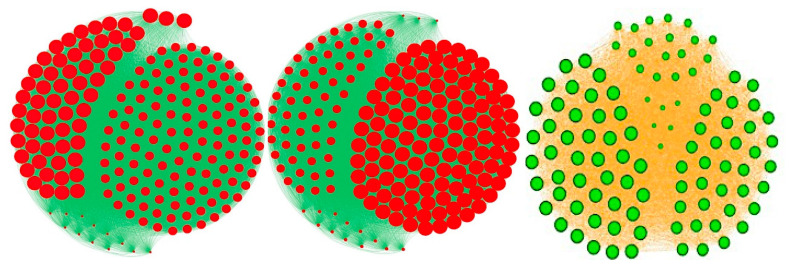
Comparison between classical types of centrality in an interaction graph, where the diameter of the point representing a node of the network is proportional to its centrality value. **Left**: eigen-centrality in an interaction graph corresponding to friendship relations in a high school. **Middle**: total-degree centrality (same interaction graph than left). **Right**: entropy centrality in a quasi-symmetric graph of friendship.

The four centralities above can be very different (Figure 6), but each has a specific interest: (i) betweenness centrality relates to the global connectivity with all nodes of the network, (ii) degree centralities correspond to the local connectivity with only nearest nodes, (iii) closeness centrality measures the proximity with other nodes using a distance on the interaction graph and (iv) eigen-centrality quantifies the ability of a node to be connected to nodes, possibly only a few, but with a high connectivity; hence, identifying important hub-relays controlling a wide sub-network.

As a complement of the classical centralities, we introduce now a new notion of centrality, called entropy centrality, taking into account the heterogeneity of the distribution of states of the neighbors of a node *i*, and not only its connectivity in the graph, and highlighting the symmetric sub-graphs having equi-distributed states in the neighborhoods of their nodes (Figure 6):

*C_i_^entropy^* = −Σ_*k* = 1,*s*_*v_k_* log_2_*v_k_*, (23)

where *v_k_* denotes the *k*th frequency among *s* (*s* = 2 in Boolean case) in the histogram of the values of states observed in the neighborhood *V_i_* of the node *i*, set of the nodes out- or in-linked to the node *i*.

## 4. Application to a Real Genetic Network

**Figure 7:**
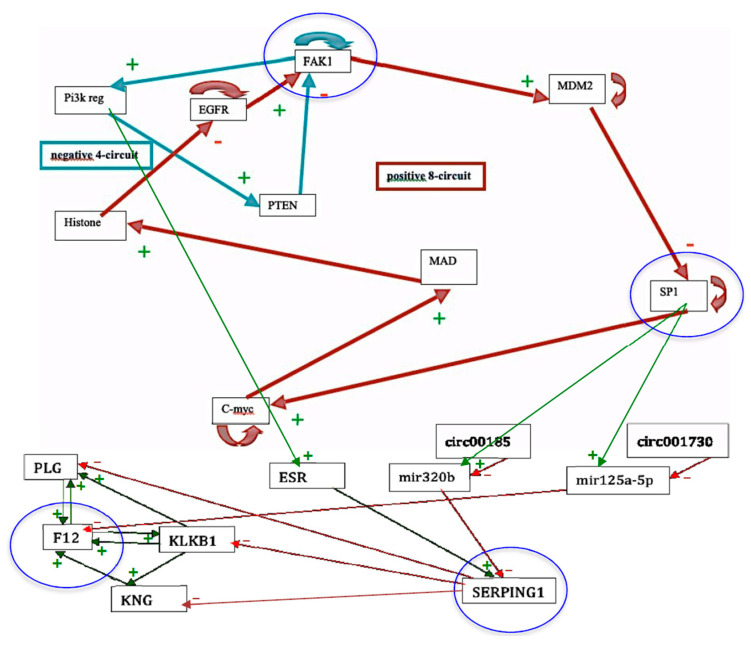
The genetic regulatory network ruling the expression of the genes involved in the angioedema disease. Genes circled in blue are those having a total-degree centrality more than five.

**Figure 8:**
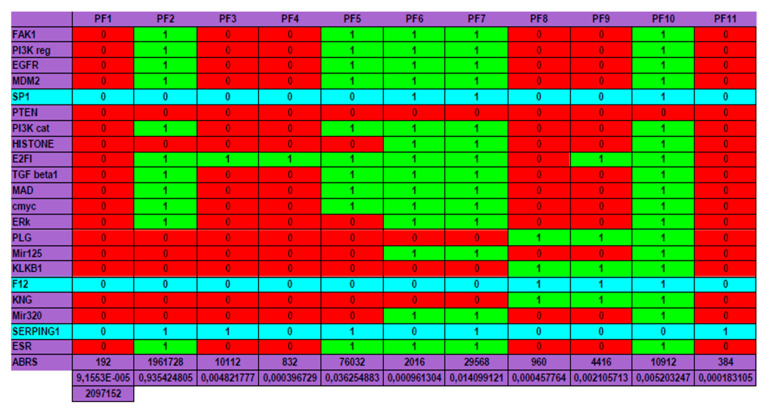
The genetic regulatory network ruling the expression of the genes involved in the angioedema disease.

The genetic regulatory network controlling kinins and kallikrein described on Figure 7 corresponds to genes involved in a familial disease, angioedema, for which there are normal profiles of genetic expression and pathologic ones, particularly in the hereditary form of the disease. By studying the connectivity of the nodes of the interaction graph of the network, we can select four critical genes having a high connectivity power with high total degree (≥5) and entropy centrality: these genes are FAK1, SP1, F12 and SERPING1. Surprisingly, the last three of them are the most involved in the etiology of the angioedema, being susceptible of mutations and/or hormonal interactions, favoring the appearance of the disease. Four are represented circled in blue in the network of Figure 7 and three are in a blue line on Figure 8.

*E_attractor_* can be evaluated by the quantity − Σ*_k =_*_1,*m*_ *ABRS*(*A_k_*)log_2_[*ABRS*(*A_k_*)], where *ABRS*(*A_k_*) is equal to the size of the attraction basin of the *k*^th^ attractor *A_k_* among the *m* attractors of the network dynamics, divided by 2*^n^*, where *n* is the number of genes of the studied network *N* (equal to 21 in the present case, by supposing that circular RNAs are absent). Then, in the present example, we have *E_attractor_* ≈ 0.4520. Hence, we have: *E* ≈ log_2_2*^n^ −* *E_attractor_* ≈ 21 − 0.4520 ≈ 20.55, which corresponds to a high value of the dynamic entropy, and consequently to a high structural stability of the genetic network *N* controlling the kinin- kallikrein system.

We can notice on Figure 8 Top that the physiologic attractor is a fixed point (PF3) whose attraction basin is made of 93.43% of the possible states of the network. The pathologic attractor corresponding to the hereditary form of the disease called angioedema is the fixed point PF10, at which there is the absence of the gene SERPING1 and the presence of the genes KLKB1 coding for kallikrein B1 and KNG coding for kininogen. The attraction basin of PF10 has a relative size equal about to 5‰ of the possible states of the network, and the observed prevalence of hereditary angioedema in the general population is reported to range from 1:10,000 to 1:150,000, without major sex or ethnic differences [87,88].

A last remark concerns the number of attractors (11 fixed points and no periodic attractor on Figure 8). A previous work [40] gives an algebraic formula allowing for the calculation of the number of attractors of a Boolean network; in their Jacobian interaction graph were two tangential circuits (Table 1): one positive of length right, (involved in the richness in attractors, as predicted by [32,33,34], and one negative of length four (responsible of the trajectory stability, as predicted by [36,89]). This predicted number (11) is the same as that calculated from the simulation of all trajectories from all possible initial conditions summarized in Figure 8, and more results both theoretical and applied to real Boolean networks can be found in more recent literature [46,47,48,49,90] showing a large spectrum of possible applications in genetic, metabolic or social Boolean networks.

## 5. Conclusions and Perspectives

The notion of dynamic entropy is common to both discrete and continuous dynamical systems and can be applied to numerous situations in biological modeling in order to interpret the dynamical behavior of complex systems having several elements in interaction, leading to many observed trajectories until their ultimate asymptotic behavior, via the attractors. Biological regulatory networks are currently widely used to design the mechanisms of these interactions at different scales, from genes and cells [68,91,92] to individuals [46,93]. Between these extreme molecular and population levels, identical functions can be concerned from different species: from paramecia, a plant-like *Bidens pilosus* to humans as memory function [49,94] or motion control [95,96,97]. Because the corresponding regulatory systems are often random, future work will be done in the framework of general random systems [98]—in which the notions of entropy, stochastic attractor (confiner) and invariant measure can help to interpret and classify the observed data in many regulatory networks (neural, genetic, metabolic, social, etc.)—and in biomedical fields for practical applications (see for example [61,99]).

## Figures and Tables

**Table 1:** Total number of attractors (here 8 in red) of two tangential circuits at the intersection of a line fixing the length L of a negative circuit (here 4) and a column fixing the length R of positive circuit (here 8) tangential to the negative one.

	**1**	**2**	**3**	**4**	**5**	**6**	**7**	**8**	**9**
**1**	**1**	**2**	**2**	**3**	**3**	**5**	**5**	**8**	**10**
**2**	**1**	**1**	**2**	**3**	**3**	**4**	**5**	**8**	**10**
**3**	**1**	**2**	**1**	**3**	**3**	**6**	**5**	**8**	**8**
**4**	**1**	**1**	**2**	**1**	**3**	**4**	**5**	**11**	**10**
**5**	**1**	**2**	**2**	**3**	**1**	**5**	**5**	**8**	**10**
**6**	**1**	**1**	**1**	**3**	**3**	**1**	**5**	**8**	**8**
**7**	**1**	**2**	**2**	**3**	**3**	**5**	**1**	**8**	**10**
**8**	**1**	**1**	**2**	**1**	**3**	**4**	**5**	**1**	**10**
**9**	**1**	**2**	**1**	**3**	**3**	**6**	**5**	**8**	**1**
